# “Embodied Psychomotor Therapy” in patients with Schizophrenia

**DOI:** 10.1192/j.eurpsy.2023.2218

**Published:** 2023-07-19

**Authors:** A. Vulpio, S. Amorosi, F. Magnani, R. Ottoni, C. Marchesi, M. Tonna

**Affiliations:** 1 Department of Medicine and Surgery, Unit of Neuroscience, University of Parma; 2 Department of Mental Health, Local Health Service, Parma, Italy

## Abstract

**Introduction:**

Evidence from contemporary research has highlighted abnormal subjective pre-psychotic experiences as an expression of schizotropic vulnerability, for which trajectories up to First Rank Symptoms have been described. Embodiment is crucial to the conceptualisation of these experiences as the distinctive feature of schizophrenic phenomena. In fact, these are embedded in precise experiential frameworks such as Diminished Self-Affection and Hyperreflexivity, which constitute *in nuce* the experience of Dis-Embodiment. The latter responds poorly to conventional therapies, thereby affecting considerably the prognosis *quoad valetudinem* of Schizophrenia.

**Objectives:**

This study is intended to explore the use of specific psychomotor therapy protocols aimed at fostering Embodiment in patients with Schizophrenia, especially by investigating its efficacy and specificity on self-perceived body disorders, on characteristic motor abnormalities and on psychopathological dimensions.

**Methods:**

The study involves the participation of 20 patients throughout 10 weekly 90-minute meetings of Embodied Psychomotor Therapy (EPT) in groups of approximately 5 participants. Despite being partially inspired by current approaches, EPT is conceived as a specific activity intended for patients with schizophrenia: each meeting combines *intersubjective coordination* activities (complex motor sequences, harmonious control of voluntary movement and movement in space, body-awareness), *intra-subjective coordination* (mirroring, demarcation and identification of one’s own boundaries, single-group dynamics), and exercises aimed at developing *motor skills* (proprioception, balance, posture, rhythm and speed). At the beginning of the activity (T0) and after 10 meetings (T1) participants will carry out self-administered and externally administered assessments, for the evaluation of motor (BMS, LOFOPT, BBS, AIMS, SRRS), psychopathological (PANSS, FBF, ABP), social functioning (SOFAS) and daily physical activity level (IPAQ) dimensions.

**Results:**

The study is still ongoing, due to limitations dictated by the Sars-CoV-2 pandemic. Preliminary results at T0 indicate a positive correlation between low levels of daily physical activity (IPAQ) and poor functioning (SOFAS). Significantly higher motor impairment with respect to the general population is also confirmed in all motor scales used. Moreover, a positive correlation between low levels of motor coordination (BMS_MC) and balance (BSS_TOT) was found together with basic symptoms related to loss of control or self-agency (FCQ_KO). Furthermore, the first results suggest an overall improvement in motor performance at T1.

**Conclusions:**

The longitudinal analysis will enable the extent of the impact of EPT on functioning, motor and psychopathological dimensions of the patients to be determined, providing useful elements for planning specific rehabilitation interventions for schizophrenia.

**Disclosure of Interest:**

None Declared

